# Effects of Printing Angle and Post-Curing Time on the Color and Translucency of 3D-Printed Temporary Restoration

**DOI:** 10.3390/biomimetics9070420

**Published:** 2024-07-10

**Authors:** Tuğba Temizci, Türkay Kölüş

**Affiliations:** 1 Department of Prosthodontics, Faculty of Dentistry, Karamanoğlu Mehmetbey University, 70200 Karaman, Turkey; 2 Department of Restorative Dentistry, Faculty of Dentistry, Karamanoğlu Mehmetbey University, 70200 Karaman, Turkey; turkaykolus@hotmail.com

**Keywords:** 3D printing, printing angle, post-curing time

## Abstract

In resins produced with a 3D printer, the printing parameters affect the properties of the restoration produced. This study examined the effect of the printing angle and post-curing time on the optical properties of temporary restorations. A total of 135 disk-shaped Formlabs temporary resins (10 × 2 mm) were produced at three different printing angles (0, 45, and 90 degrees) and post-cured for three different times (20, 40, and 60 min) (n = 15). Color and translucency measurements were taken for each group with a spectrophotometer (VITA Easyshade V). The ΔE values between printing angles and curing times influence each other. The highest color change was observed in the groups produced with a 90° printing angle. Considering the post-curing times, the highest color change was observed in the groups cured for 40 min. Increasing the curing time from 20 to 40 min decreases the translucency, whereas further increasing the curing time does not significantly affect the translucency. In terms of the impact on the translucency caused by the printing angles, 0° exhibited a lower translucency compared to other printing angles. During the 3D printing of temporary prostheses, both printing angles and post-curing times can affect their optical properties.

## 1. Introduction

Computer-aided design (CAD) and computer-aided manufacturing (CAM) have changed modern dental clinical workflows [1]. The even newer and more popular additive manufacturing (AM) technology, also known as 3D printing [1], enables the manufacture of many restorations with less expensive equipment, reducing the material waste and production time [2,3]. AM has made great progress in restorative dentistry, becoming an effective method for addressing clinical needs [3,4,5]. This technology has a wide range of applications, including surgical guides, anatomical models, occlusal splints, temporary and permanent prosthetic restorations, orthodontic appliances, and attachments [6]. It is used to produce prostheses from polymers, ceramics, and metals [7]. These advantages demonstrate how 3D printing has revolutionized dentistry, offering more precise and personalized solutions to patients; 3D-printed resins have been tested for temporary and medium-term usage (up to two years) and are appropriate for crowns, inlays, onlays, and bridges [8,9]. AM’s key faults include anisotropy and a low filler content, which will affect the physical properties of printed resin-based structures [10].

Unlike subtractive manufacturing, layer-by-layer production in additive manufacturing prevents wear issues associated with milling or drilling and allows for the easy printing of complex geometries [11]. Classified by ASTM (American Society for Testing and Materials) as one of the additive manufacturing technologies under vat photopolymerization, SLA (stereolithography) technology is the preferred method in dentistry due to its superior resolution, smooth surface quality and adequate z-axis strength [12,13,14]. In SLA technology, a laser beam is focused on a photosensitive liquid resin, curing it layer by layer. When one layer of resin is fully cured, the platform moves vertically to cure the next layer. The process is repeated until the object is fully formed. However, a limitation of SLA is the necessity of support structures for object fabrication. On the other hand, the advantages of SLA include temperature resistance and the ability to produce complex geometries [13], which is why SLA technology was chosen for our study.

During the 3D printing process, several parameters must be controlled. The quality of the printed material is affected by the depth and degree of polymerization, layer thickness, shrinkage between layers, and the intensity and angle of the light source [15,16,17,18]. The number of layers varies according to the printing direction, and shrinkage might occur between layers [18]. It is critical that we properly comprehend every factor that can influence the quality of a prosthesis in 3D printing [11,19].

After the object has been printed using photopolymerization, unreacted initiators and monomers remain. To complete the polymerization, a post-processing technique involving further irradiation is required. This is achieved through the use of polymerization equipment with a wavelength range that is compatible with the polymerization process [10]. The post-printing polymerization process is essential to the quality of the finished product.

When the long-term use of temporary restorations is necessary, particularly in the anterior region, patients’ aesthetic expectations and demands will increase. Therefore, the optical properties of temporary restorations are important considerations for clinicians. The most critical factors affecting the aesthetics of dental restorations are considered to be the color and translucency of a material [20]. Translucency is the ability of a colored material to allow the underlying background to be seen [21]. Incident light undergoes reflection, absorption, scattering, and transmission within the dental material, and translucency is determined by the interaction of these events [22].

Moreover, 3D-printed resins offer a wide range of tonal variability [23]. The chemical composition of the material [24,25], type of filler [26,27,28], photoinitiators [29], and pigments [30,31], as well as the layer thickness [21] and other aspects related to the design and production stages of the sample [25,32,33], washing [34,35], and post-curing protocols [9,33,36,37], can affect the quality of 3D-printed restorations. The majority of studies on 3D-printed polymers have concentrated on their physical and mechanical properties, dimensional accuracy, and durability [3,4,38,39,40]. Gaining sufficient knowledge about the color perception, behavior, and appearance of dental resins, obtained through understanding their optical properties, can assist clinicians in selecting the appropriate material and shade to achieve a more natural appearance in dental restorations.

The printing parameters significantly influence the final material properties [5]. Specifically, the printing angle is a crucial parameter that can address the anisotropy and physical weaknesses of the printed material resulting from the layering production technique [12]. It has been demonstrated that the printing orientation affects the mechanical properties [4,39,40,41] and printing accuracy [42] of 3D-printed restorative resins. There is limited scientific knowledge about the optimal printing process and post-curing techniques required to achieve restorations with sufficient aesthetics from 3D-printed resins [8,26]. Therefore, this study aims to evaluate the effect of the printing angle and post-curing time on the optical properties of temporary restorations. The first hypothesis of the study is that the printing angle will affect the optical properties. The second hypothesis is that the post-curing time will also affect the optical properties.

## 2. Materials and Methods

For two-way ANOVA, the power analysis conducted using G*Power software (Ver. 3.0.10) with parameters of 80% power, 0.05 alpha error probability, and 0.303 effect size determined that a minimum sample size of 135 was required.

**Figure 1:**
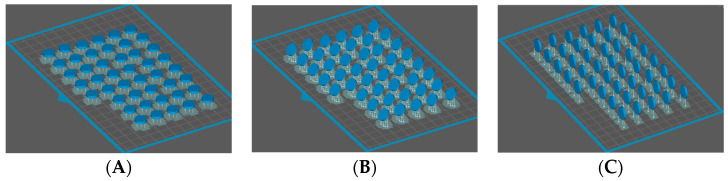
Specimen preparation: (**A**) 0 degree printing angle, (**B**) 45 degree printing angle, (**C**) 90 degree printing angle.

**Figure 2:**
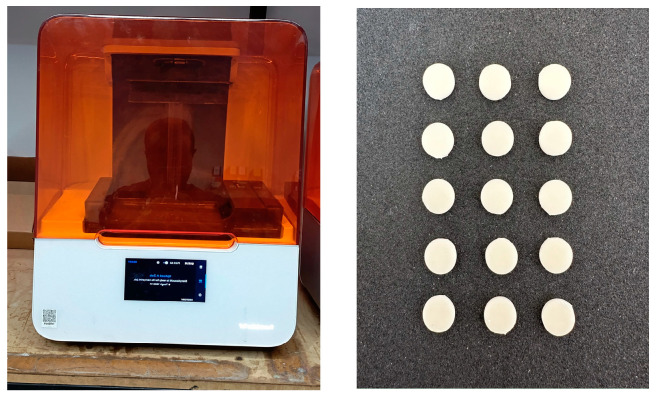
Specimens printed using 3D Form 3B printer.

For the study, Formlabs Temp 3D printing temporary resin in A2 shade was selected. A total of 135 disk-shaped samples (10 × 2 mm) were designed using CAD software and transferred to the printer as STL files. The samples were produced using SLA technology on a Form 3B printer (Formlabs, Somerville, MA, USA) with a layer thickness of 50 μm at three different printing angles (0, 45, and 90 degrees). The preparation of the specimens is shown in Figure 1. The samples at each printing angle were cured in a FormCure device (Formlabs Inc., Somerville, MA, USA) for three different post-cure durations (20, 40, and 60 min) (n = 15) (Figure 2).

A low-speed rotary tool was used to remove the support structures, and water cooling was used to grind the specimens with abrasive paper (up to 1200 grit) on both sides. The thickness of the specimens was measured using a digital caliper (Mitutoyo, Europe GmbH, Germany) with an accuracy of 0.01 mm, resulting in a final thickness of 2.00 mm ± 0.01 mm. Following a 5 min ultrasonic cleaning, the specimens were immersed in distilled water at 37 °C for 24 h.

### 2.1. Color Measurement

**Figure 3:**
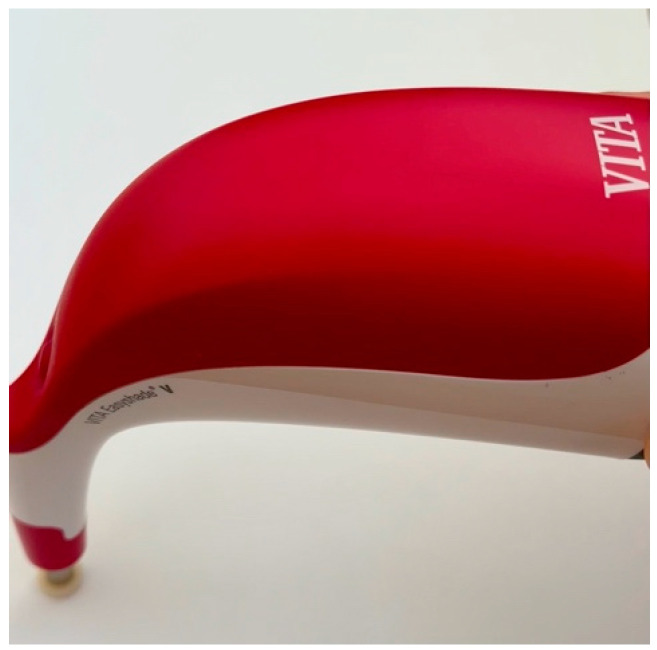
Color and translucency measurement with spectrophotometer.

The colors of the samples were determined using a spectrophotometer (VITA Easyshade V, VITA Zahnfabrik, KG, Germany) with the CIEDE2000 (ΔE00) formula. The spectrophotometer tip was placed directly on the specimen surfaces. A single operator conducted color measurements on a white background. The color of each sample was measured three times, and the average L, a, and b values were recorded (Figure 3).

(1) $$\Delta E_{00}=\sqrt{\left({\frac{\Delta L^{’}}{k_{L}S_{L}})}^{2}+({\frac{\Delta C^{’}}{k_{C}S_{C}})}^{2}+({\frac{\Delta H^{’}}{k_{H}S_{H}})}^{2}+R_{T}(\frac{\Delta C^{’}}{k_{C}S_{C}})(\frac{\Delta H^{’}}{k_{H}S_{H}}\right)}$$

In this study, the parametric factors of the ∆E00 were fixed to 1. If the ∆E00 value did not exceed 2.25, then the hue change was considered clinically acceptable [11].

### 2.2. Translucency Measurement

The translucency parameter (TP) of each specimen was calculated by determining the color difference between the specimen and the black and white standards, using the following equation [43]:

TP = [(L*B − L*W)^2^ + (a*B − a*W)^2^ + (b*B − b*W)^2^]^1/2^, (2)

where L*B, a*B, and b*B were measured against the black background and L*W, a*W, and b*W were measured against the white background. Translucency discrepancies were ultimately analyzed using published data for 50:50% translucency perceptibility (TPT00 = 0.62) and acceptability (TAT00 = 2.62) criteria [44].

### 2.3. Statistical Analysis

The Shapiro–Wilk test determined that the data were normally distributed, while Levene’s test confirmed that the variances were homogeneous (*p* ≤ 0.05). The data were evaluated using a two-way analysis of variance (SPSS 20.0 software; IBM, Chicago, IL, USA), followed by a Tukey honest post hoc test to identify differences between groups. The statistical significance level was set at *p* < 0.05.

## 3. Results

### 3.1. Color Change Results

According to the 2-way ANOVA results, the printing angle affects the color change between post-curing times, and the post-curing time affects the color change between printing angles (*p* < 0.05). The highest color change was observed in the groups produced with a 90° printing angle. Also, in the group produced with a 90° printing angle, the color change (ΔE_00_) between the curing times of 20–40 and 40–60 min was higher than 2.25 and was found to be clinically unacceptable (Table 1).

According to the results of the post hoc Tukey test comparing the printing angles, there was no significant difference in the average color difference between 0° and 45°, while the color difference between 0° and 90° and 45° and 90° was statistically significant.

The highest color change was observed when the post-cure time was 40 min. The color difference between those produced with a 45° and a 90° printing angle cured for 40 min was higher than 2.25 and was found to be clinically unacceptable (Table 2).

According to the results of post hoc multiple comparisons, there is no statistical difference between the amount of ΔE between 40 and 60 min and 20 and 60 min. The change between other times statistically affected the ΔE (*p* ≤ 0.05). There was no statistical difference between the amounts of ΔE between the printing angles (*p* ≥ 0.05) (Table 3).

### 3.2. Translucency Results

The translucency values of different configuration groups are shown in Table 4. Regardless of the production angle or the length of the post-curing time, the translucency values of the samples were above the clinically accepted value of 2.62.

According to the post hoc Tukey test of a two-way ANOVA, the post-curing time has a significant effect on translucency values. When the post-curing time is increased from 20 to 40 min, the translucency decreases (*p* ≤ 0.05), but it does not change when the post-curing time is increased from 40 to 60 min (*p* ≥ 0.05). While there is no difference between 45° and 90° in terms of translucency (*p* ≥ 0.05), 0° has lower translucency than the other angles (*p* ≤ 0.05) (Table 5).

## 4. Discussion

This study investigated the effect of the printing angle and post-curing time on the color and translucency of temporary restorations produced with a 3D printer. The first hypothesis established at the beginning of the study was accepted: color and translucency values can be affected by the printing angle. The second hypothesis of the study was also accepted, indicating that the post-curing time affects the optical properties.

In the case of the long-term use of temporary restorations, color matching between natural teeth and restorations is important [4]. In order to produce predictable dental restorations and to maximize these properties in material development, it is of the utmost importance to gain an understanding of how 3D-printed materials react, in terms of color and translucency, to changes in the printing process. Visual color difference thresholds are an accepted quality control approach in dentistry [11]. Lee et al. [45], in their study on the effect of the printing angle on color stability, found that samples produced at a 0° printing angle exhibited less color change compared to those produced at 45° and 90° angles. In contrast, Castro et al. [46] found no effect from the printing angle on color change. Similarly, our study demonstrated the highest color change in samples produced at 90°. Espinar et al. [47] also found statistically significant differences in CIELAB coordinate values between resins printed at 0° and 90°. Similarly, our study shows that different printing angles (0°, 45°, and 90°) affect both color and translucency. Clinicians should exercise caution in selecting materials and shades for restorations produced using 3D printers. Optical characteristics are connected with light absorption and scattering on the surface and in the surrounding environment [22,48]. Light scattering has a strong correlation with perceived hue and translucency [47,48,49]. When it comes to resin-based composites, scattering is primarily governed by particle shape and size, whereas absorption is dependent on the presence and type of the resin matrix and coloring pigments [49,50,51]. The translucency of resin-based dental restorative materials is dependent upon the refractive indices (n) of the organic matrix and the filler material [52]. However, printed samples typically consist of several layers, and the translucency discrepancies are presumably caused by the orientation of overlapping layers during the printing process [53]. Each layer and interface within a multilayered sample is responsible for reflecting and transmitting light [53]. Light can scatter and/or be absorbed within the layers, and interfaces between layers with different refractive indices can reflect and transmit light [54]. This could explain the varying degrees of translucency differences depending on the printing direction.

It has been established that the varied monomer levels in 3D printing resin formulations affect mechanical properties [55]. Furthermore, 3D printing resins show unique anisotropy in their mechanical properties, defined as distinct behaviors for different printing orientations [54]. This property–composition relationship and anisotropic behavior are consistent with our findings on color and translucency. Consequently, the orientation of the printing affects not only the mechanical behavior of resin-based 3D-printed restorations but also their aesthetic appearance.

Tayaheri et al. [16] investigated the degree of conversion between samples printed at 0° and 90° angles. They discovered that the polymerization at the “top” of the 3D-printed rods (closer to the printing platform) was slightly higher than at the “base”. Because of the square form of the color measuring region on the samples’ flat surface, these overpolymerized layers will be present in the 0° samples but not in the 90° samples. The existence or absence of layers with greater degrees of conversion in the sample measurement area, depending on the printing angle, can explain changes in light interaction [53], as well as differences in the final color and translucency of the printed resin. Further research may be conducted to gain a deeper understanding of the relationship between color and translucency, as well as the extent of conversion of 3D-printed resins.

Kim et al. [11] conducted a study examining color change at different post-cure times (15, 30, 60, 90, and 120 min) and found that as the curing time increased, the color darkened. Lee et al. [41], in their research on the effect of post-cure times (0, 5, 10, and 20 min) on color stability, observed that longer post-cure times resulted in better color stability. Soto-Montero [42] demonstrated that post-curing times of 5–10 min did not significantly affect the color acceptability of the material. After printing, light-curable resins undergo post-curing processes to cross-link and polymerize any remaining uncured monomers [56]. The equipment and application times used in curing processes can vary between companies. When exposed to adequate light sources, the resin’s terminal aliphatic C-C linkages are broken and converted into primary C-C covalent bonds between methacrylate monomers. Higher polymerization often results in improved mechanical characteristics and biocompatibility, while lowering remaining monomers [56,57]. As a result, post-curing plays an important role in the final qualities of 3D-printed prosthetics.

The greater color difference between the 20–40 min interval and the 20–60 min interval can be attributed to the effects of prolonged ultraviolet (UV) irradiation. UV light is used in the post-curing process to enhance the mechanical properties of 3D-printed resins, but its duration and intensity can significantly impact other properties, including color and translucency [58].

Depending on parameters such as the printing, washing, and post-curing processes, 3D-printed prosthetics can yield different outcomes [59]. Studying how the characteristics of 3D-printed objects evolve over time post-curing can offer essential insights for clinicians and dental technicians engaged in prosthetic manufacturing. 

It is well known that changes in color tone with curing time are primarily attributed to the photoinitiator [31,60]. The appropriate combination of photoinitiator and co-initiator, along with the exposure time to the light source, can induce desired changes in color tone. These processes not only improve biocompatibility but also enhance the mechanical strength [61]. For instance, when using TPO and BAPO as photoinitiators in the polymerization process, there tends to be an increased yellowing effect [31,62]. These photoinitiators polymerize quickly, and the temperature rise during polymerization can lead to the formation of colored peroxides, resulting in noticeable yellowing [62]. Additionally, the characteristics of the curing equipment’s light source can also influence the final color tone of the 3D-printed resin.

During 3D design, changing the orientation of objects often speeds up printing [63], prevents printing supports in unwanted areas of the object, or allows for the simultaneous production of multiple objects. Therefore, we aimed to compare production at different printing angles. Some studies have reported on the effects of different post-cure devices [33,37,64] and conditions [7,11,56,65,66] on the mechanical and biological properties of printed resins, but studies examining optical properties are relatively scarce.

Accessing detailed information about the components used in 3D-printed resins is challenging due to intellectual property rights, which is a limitation of this study. This limitation poses a significant barrier to the comprehensive evaluation and understanding of these materials within our study. Additionally, a clinical limitation of this study is the irregular morphology of temporary crowns and bridges, which are not uniformly disk-shaped. This irregularity potentially leads to varying behaviors in 3D-printed resins in terms of color and translucency. Consequently, this highlights the necessity for further research to thoroughly investigate these aspects and understand the full implications of using 3D-printed resins in dental applications.

## 5. Conclusions

The printing angle significantly impacts the ΔE values between curing times, and the curing time similarly affects the ΔE values between different printing angles. Specifically, increasing the curing time from 20 min to 40 min results in a decrease in translucency, while further extending the curing time does not have a significant impact on the translucency. Among the various printing angles, the 0° angle exhibited a lower translucency compared to other angles.

These findings indicate that both the printing angle and post-curing times play crucial roles in determining the optical properties of temporary prostheses produced with 3D printing technology. Consequently, optimizing UV curing protocols is essential in achieving consistent and desirable color outcomes. Further research is necessary to comprehensively understand these interactions and refine the processes for improved clinical applications. 

## Figures and Tables

**Table 1:** Means and standard deviations of color difference between post-curing time points (ΔE_00_).

Printing Angle	Post-Curing Times between Which ΔE Was Measured	Mean	Std. Deviation	N
0°	20–40 min	2.2200	1.10342	15
	20–60 min	1.5800	0.87820	15
	40–60 min	1.5387	1.52961	15
45°	20–40 min	2.1960	1.55907	15
	20–60 min	1.4833	0.83184	15
	40–60 min	1.9347	1.45855	15
90°	20–40 min	3.2693	1.28991	15
	20–60 min	2.1320	1.37863	15
	40–60 min	2.3033	1.18075	15

**Table 2:** Means and standard deviations of color difference between printing angles (ΔE_00_).

Post-Curing Time	Angles between Which ΔE Was Measured	Mean	Std. Deviation	N
20 min	0°–45°	1.9747	1.03028	15
	45°–90°	1.9473	1.04465	15
	0°–90°	1.6567	1.03923	15
40 min	0°–45°	2.1447	1.72213	15
	45°–90°	2.3313	2.10046	15
	0°–90°	1.7067	1.08973	15
60 min	0°–45°	1.1847	0.81700	15
	45°–90°	2.1073	1.27027	15
	0°–90°	1.9873	1.32617	15

**Table 3:** Results of multiple-comparison post hoc test.

(I) Curing Times at Which ΔE Was Measured	(J) Curing Times at Which ΔE Was Measured	Mean Difference (I–J)	Std. Error	Sig.	95% Confidence Interval	
					Lower Bound	Upper Bound
20–40 min	40–60 min	0.6362 *	0.26793	0.050	0.0008	1.2717
20–60 min	20–40 min	−0.8300 *	0.26793	0.007	−1.4655	−0.1945
40–60 min	20–60 min	0.1938	0.26793	0.750	−0.4417	0.8292
**(I) Printing Angles at Which ΔE Was Measured**	**(J) Printing Angles at Which ΔE Was Measured**					
0°–45°	45°–90°	−0.3607	0.27966	0.404	−1.0240	0.3026
45°–90°	0°–90°	0.3451	0.27966	0.435	−0.3182	1.0084
0°–90°	0°–45°	0.0156	0.27966	0.998	−0.6477	0.6788

* indicates statistically significant differences (*p* < 0.05).

**Table 4:** Means and standard deviations of translucency.

Printing Angle	Post-Curing Time	Mean	Standard Deviation	n
0°	20 min	4.8833	0.46449	15
	40 min	4.5400	0.45902	15
	60 min	4.8107	0.48075	15
45°	20 min	5.6767	0.41636	15
	40 min	5.4513	0.49666	15
	60 min	5.3847	0.28168	15
90°	20 min	5.8767	0.49928	15
	40 min	5.1613	0.52565	15
	60 min	5.4973	0.37167	15

**Table 5:** Post hoc Tukey test results comparing the effects of post-curing times and printing angles on translucency.

(I) Post-Cure Time	(J) Post-Cure Time	Mean Difference (I–J)	Std. Error	Sig.	95% Confidence Interval	
					Lower Bound	Upper Bound
20 min	40 min	0.4280 *	0.09482	0.000	0.2031	0.6529
40 min	60 min	−0.1800	0.09482	0.143	−0.4049	0.0449
60 min	20 min	−0.2480 *	0.09482	0.027	−0.4729	−0.0231
**(I) Printing Angle**	**(J) Printing Angle**					
0°	45°	−0.7596 *	0.09482	0.000	−0.9844	−0.5347
45°	90°	−0.0076	0.09482	0.997	−0.2324	0.2173
90°	0°	0.7671 *	0.09482	0.000	0.5422	0.9920

* indicates statistically significant differences (*p* < 0.05).

## Data Availability

The data presented in this study are available on reasonable request from the corresponding author.
